# Gr-1^+^CD11b^+^ Myeloid-Derived Suppressor Cells: Formidable Partners in Tumor Metastasis

**DOI:** 10.1002/jbmr.154

**Published:** 2010-06-22

**Authors:** Li Yang, Claire M Edwards, Gregory R Mundy

**Affiliations:** 1 Department of Cancer Biology, Vanderbilt University Medical CenterNashville, TN, USA; 2 Laboratory of Cancer Biology and Genetics, Center for Cancer Research, NCI, National Institutes of HealthBethesda, MD; 3 Vanderbilt Center for Bone Biology, Department of Cancer Biology, Vanderbilt University Medical CenterNashville, TN, USA; 4 Vanderbilt Center for Bone Biology, Department of Medicine/Clinical Pharmacology, Vanderbilt University Medical CenterNashville, TN, USA

**Keywords:** myeloid-derived suppressor cells, GR-1, CD11B, myeloma, metastasis, osteoclasts

## Abstract

The growth and metastasis of solid tumors not only depends on their ability to escape from immune surveillance but also hinges on their ability to invade the vasculature system as well as to induce the formation of new blood vessels. Gr-1^+^CD11b^+^ myeloid-derived suppressor cells (MDSCs), overproduced in tumor-bearing hosts, contribute significantly to all these aspects. They also have a potential role in the osteolysis associated with bone metastases. They are formidable partners in tumor metastasis. © 2010 American Society for Bone and Mineral Research.

## Introduction

Metastasis is the cause of cancer mortality. The growth and metastasis of solid tumors not only depend on their ability to escape from immune surveillance but also hinge on their ability to invade the vasculature system as well as to induce the formation of new blood vessels. Thus tumor blood vessels are promising therapeutic target in the treatment of neoplastic diseases. The approval of bevacizumab, a humanized monoclonal antibody that recognizes and blocks vascular endothelial growth factor A (VEGFA), by the US Food and Drud Administration as a first-line therapy for metastatic colorectal cancer validates the ideas that VEGF is a key mediator of tumor angiogenesis and that blocking angiogenesis is an effective strategy to treat human cancer.(1–3)

The microenvironment of solid tumors contains regions of poor oxygenation and high acidity. Growing evidence from clinical and experimental studies points to a fundamental role for hypoxia in metastatic progression. Prolonged hypoxia increases genomic instability, genomic heterogeneity, and epigenetic alteration and thus may act as a selective pressure for tumor cell variants.(4)

**Fig. 1:**
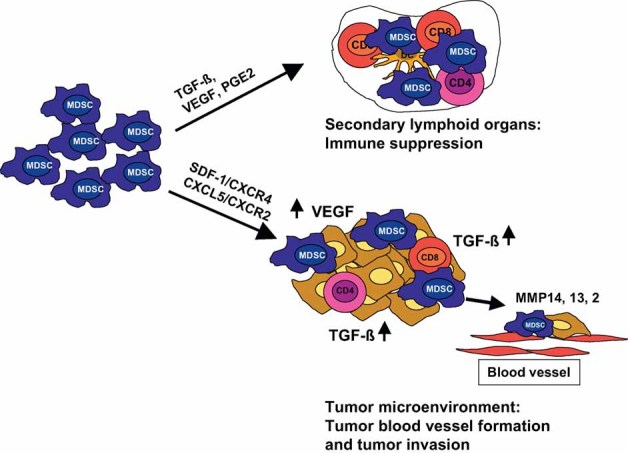
Formidable partners in tumor metastasis: MDSCs suppress host immune surveillance through the production of multiple immune suppressive cytokines, including TGF-β, VEGF, and PGE2. MDSCs also infiltrate the tumor microenvironment and contribute to tumor vasculature formation. In addition, these immature myeloid cells produce large quantities of TGF-β and a variety of MMPs that promote tumor invasion.

One of the responses of the tumor host to hypoxic conditions is an inflammation reaction. This is regulated through hypoxia-inducible factor 1α (HIF-1α) and NF-κB–mediated chemokine and cytokine secretion,(5) resulting in the infiltration of a variety of host-derived inflammatory cells into tumor tissues.(6) These host cells create an environment that favors tumor progression. They provide growth factors, proangiogenic factors, proteases, and adhesion molecules that facilitate tumor cell proliferation, angiogenesis, invasion, and metastasis.(7,8) Of the chemokines and chemokine receptors in inflammatory cell recruitment, stromal-derived factor 1 (SDF-1 or CXCL12) is considered one of the key regulators of hematopoietic stem and progenitor cell trafficking between the peripheral circulation and targeted tumor tissues. SDF-1 mediates its effects on chemotaxis through its receptor, CXCR4, which is highly expressed on putative stem and progenitor cells.(7) Another chemokine, CXCL5, is significantly elevated in response to deleted or diminished transforming growth factor β (TGF-β) signaling in tumor epithelial cells. This chemokine was implicated initially in the recruitment of neutrophils in the inflamed lung. Recent studies showed that CXCL5, signaling through the CXCR2 receptor, was responsible for the recruitment of Gr-1^+^CD11b^+^ cells into the breast carcinoma tumor microenvironment with deletion of TGF-β signaling(9) (Fig. 1).

## Gr-1^+^CD11b^+^ Myeloid-Derived Suppressor Cells

Gr-1^+^CD11b^+^ cells or MDSCs are significantly overproduced in the bone marrow and spleens of tumor-bearing mice. There are high numbers of these cells in peripheral blood of tumor hosts, including cancer patients. MDSCs express CD11b, a marker for myeloid cells of the macrophage lineage, and a marker for granulocytes, Gr-1; thus they are called Gr-1^+^CD11b^+^ cells. MDSCs have been demonstrated to be immune suppressive since the 1980s. They inhibit natural killer (NK), B, and T cells through the production of arginase and reactive oxygen species; they also inhibit functional maturation of dendritic cells and promote type II macrophage development and thus represent one mechanism of tumor escape from immune system control and compromise the efficacy of cancer immunotherapy.(10,11) The immune suppression by these cells can be both a systemic effect on secondary lymphoid organs and a local effect within the tumor microenvironment.

There are two major subpopulations of these cells: mononuclear cells (precursors for macrophages) and low-density polymorphonuclear cells (immature neutrophils). Both populations suppress antigen-specific T-cell responses, but through distinct effector molecules and signaling pathways.(12) Recently, MDSCs were found to directly disrupt the binding of specific peptide–major histocompatibility complex (pMHC) dimers to CD8-expressing T cells through nitration of tyrosines in a T-cell receptor (TCR)–CD8 complex. This process makes CD8-expressing T cells unable to bind pMHC dimers and to respond to the specific peptide, although they retain their ability to respond to nonspecific stimulation.(13)

Interestingly, MDSCs produce large quantities of TGF-β. TGF-β is known to inhibit immune surveillance mechanisms in the tumor host.(14) Publications from different laboratories, including our own, demonstrate that Gr-1^+^CD11b^+^ immature myeloid cells are a major source for high levels of TGF-β in the tumor host.(9,15,16) This induction of TGF-β by myeloid cells plays a greater role in suppressing the immune response than production of TGF-β by the tumor itself because blocking production by these myeloid cells abrogated the suppression, even though the TGF-β production by the tumor itself was not affected.(15) TGF-β markedly and directly suppresses the transcription of genes encoding multiple key proteins of the cytotoxic program of CD8^+^ cytotoxic T lymphocytes (CTLs) such as perforin and granzymes, cytotoxins that act through the granule exocytosis pathway.(17) This inhibition of CD8^+^ CTLs is mediated by Gr-1CD11b myeloid cells through the production of TGF-β. TGF-β also alters the polarization of the CD8^+^ cells in tumor-bearing mice, resulting in elevated interleukin 17 (IL-17), which suppressed apoptosis of tumor cells.(18) TGF-β, coordinated with IL-21, induces CD4^+^CD25^+^ regulatory T cells that counterbalance the effect of IL-6 that promotes proinflammatory IL-17-producing T cells.(19) In addition, TGF-β is responsible for CD4^+^CD25^+^ regulatory T-cell inhibition of NK cell functions.(20)

## MDSCs in the Tumor Microenvironment: More Than Immune Suppression

Despite the data defining the overproduction of MDSCs in tumor hosts and their depressive effect on host immune surveillance, it is not clear whether these cells infiltrate into the tumor microenvironment and whether they have direct interactions with tumor cells. If they do, what molecular mechanisms mediate their recruitment into the tumor microenvironment? We found that MDSCs infiltrate into tumors and promote tumor angiogenesis by expressing high levels of matrix metalloproteinase 9 (MMP9) and by directly incorporating into tumor endothelium.(21) Furthermore, MDSCs produce large quantities of TGF-β and a number of MMPs, including MMP13, MMP14, and MMP2. They migrate to the invasive front of the tumors, facilitating tumor cell invasion and metastasis(9) (Fig. 1). Recently, MDSCs have also been implicated in tumor refractoriness to anti-VEGF treatment.(22) Bv8 (prokineticin 2), expressed in the bone marrow, modulates mobilization of CD11b^+^Gr-1^+^ cells from the bone marrow during tumor development and also promotes angiogenesis locally.(23) These studies suggest that tumor-infiltrating bone marrow–derived MDSCs change the dynamics in the primary tumor microenvironment and result in alterations in the signaling cascade in tumor cells, promoting tumor cell invasion and metastasis. This work points out the important participation of host cells and strongly supports the “seed and soil” theory.

Several lines of evidence make the contribution of Gr-1^+^CD11b^+^ to tumor metastasis particularly interesting: (1) These cells are overproduced in tumor hosts that include cancer patients with a variety of tumors. (2) MDSCs are composed of immature myeloid cells at the early stages of differentiation. They are different from terminally differentiated tumor-associated macrophages (TAMs), identified as Mac-1 (CD11b) and F4/80^+^, which have been shown to promote tumor progression and metastasis through elevated CSF-1 production and enhanced epidermal growth factor (EGF) signaling in cancer cells.(24) Similarities between TAMs and these immature myeloid cells were noticed from profiling work,(25) but differences between the two populations also were evident. For example, myeloid suppressor cells produce high levels of TGF-β1, whereas TGF-β1 expression in TAMs was restricted to unstimulated TAMs and was not increased further by M2-biasing cytokines.(25) M2 refers to a subclass of macrophages based on their preferential secretion of TGF-β, IL-4, and IL-10 compared with M1 macrophages, which are activated to produce nitrogen oxide, IL-12, interferon-γ (IFN-γ). MDSCs are also different from two other cell types in the tumor microenvironment—neutrophils and mast cells, which express Gr-1 but not CD11b. (3) MDSCs interact with other host immune cells, including T, B, and NK cells. These cells may dictate to the tumor microenvironment that a shift from inflammation/immune response to anti-inflammatory/immune-suppressive responses (Th1/Th2-like cytokine shift) may be responsible in the metastatic liver milieu, as reported previously.(26) It is unclear whether systemic immune suppression and direct participation in tumor progression are two different properties or different manifestations of the same process.

## TGF-β, a Key Regulator in the Interplay of Cancer Cells and Bone Marrow-Derived Inflammatory Cells

In addition to the suppressive effect in the immune system, TGF-β is also one of the important regulators in inflammatory reactions that orchestrate the tumor microenvironment. TGF-β affects the expression of chemokine and chemokine receptors such as CXCL5, SDF-1, and CXCR4. TGF-β also mediates NF-κB signaling, the master regulator of inflammation reaction. Tumor-infiltrating RANKL (receptor activator of NF-κB ligand)–expressing cells activate nuclear IKKα and inhibit the transcription of tumor metastasis suppressor Maspin, thereby promoting tumor metastasis.(27) TGF-β1 negatively regulates NF-κB activation in the gut through Smad7.(28) Inflammation induced by *Helicobacter pylori* infection in SMAD3-deficient mice caused the development of colon cancer.(29) Furthermore, TGF-β crosstalks with inflammatory pathways through the modulation of IL-1.(30) In addition to epithelial cells, TGF-β signaling in stromal cells has significant effects on tumor development and growth. Loss of the TGF-β type II receptors in fibroblasts promotes mammary carcinoma growth and invasion through upregulation of TGF-α–, macrophage-stimulating protein (MSP)– and hepatocyte growth factor (HGF)–mediated signaling networks.(31)

Recent work from our laboratory suggests that TGF-β is a key regulator in the interplay of cancer cells and bone marrow–derived MDSCs. Diminished TGF-β signaling in breast tumor cells resulted in the recruitment of MDSCs to the invasive front. This is regulated through increased CXCL5/CXCR2 and SDF-1/CXCR4 chemokine signals(9) (Fig. 1). In turn, these myeloid cells produce large quantities of MMPs and TGF-β1, thus promoting tumor invasion and metastasis. Our observation is supported by a recent publication in which C–C chemokine receptor type 1 (CCR1)–positive myeloid cells (CD34^+^) are shown to be recruited to colon cancers with deletion of Smad4 and promote tumor invasion.(32) Indeed, inflammatory cells (positive for CD45 and BM8, a pan-macrophage marker) have been observed in head-and-neck tumors lacking TGF-β signaling.(33) In TGF-β1-deficient mice, inflammation causes precancerous lesions to progress to colon cancer.(34)

In the distant premetastatic lung, TGF-β is one of the factors produced by tumor cells responsible for the production of the chemoattractants S100A8 and S100A9, which attract Mac1^+^ myeloid cells.(35) Through this mechanism, tumor cells also activated mitogen-activated protein kinase (MAPK) p38 to acquire migratory activity with pseudopodia for invasion (invadopodia).(35) In addition, TGF-β also induces angiopoietin-like 4 expression in cancer cells that are about to enter the circulation. This disrupts vascular endothelial cell-cell junctions, increases the permeability of lung capillaries, and facilitates the transendothelial passage of tumor cells. This mechanism seems to be important for tumor cell retention in the lungs but not in bone.(36)

## MDSCs and Bone Metastasis

Hematopoietic progenitors, or MDSCs, play an important role in the progress of metastasis at any site and certainly in the bone marrow. Therefore, they are likely important in the progression of multiple myeloma and other tumors that frequently metastasize to bone, including breast cancer, prostate cancer, and lung cancer. Since MDSCs have a cell surface phenotype that overlaps with that of progenitors in the osteoclast lineage, this suggests that they also may have a capacity to develop into osteoclasts at this site. Our group has examined their role in the progression of the osteolytic bone disease associated with multiple myeloma. Myeloma bone disease is characterized by osteoclast activity adjacent to myeloma deposits. Nothing is known of the origin of osteoclasts in either patients or preclinical murine models of myeloma. We do not know where they come from or what their precursors are. However, it is clearly likely to be an issue of importance. The molecular signals that pass between osteoclast precursors and myeloma cells in the bone microenvironment are likely critical to progression of the disease, raising the possibility that myeloma cells may influence MDSC differentiation and/or function.

**Fig. 2:**
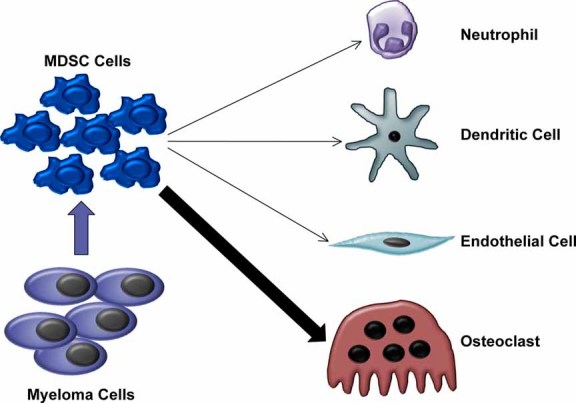
Potential role of MDSCs in myeloma bone disease. MDSCs are known to have the capacity to differentiate into neutrophils, dendritic cells, and endothelial cells. We propose that myeloma cells may stimulate MDSCs to differentiate into osteoclasts and contribute to the osteolytic bone disease.

We have studied this in the 5T model of myeloma. The 5T model of myeloma was described originally by Radl and colleagues.(37,38) They found that 1 in 200 C57Bl mice of the KaLwRij strain developed myeloma spontaneously and that the myeloma cells can be passaged by tail vein injection from mouse to mouse. This reproduces the human disease remarkably, with all the regular characteristics of myeloma, including tumor growth within the bone marrow and the development of an osteolytic bone disease. In this murine model, MDSCs were increased in the bone marrow and spleen of myeloma-bearing mice and correlated with progression of the disease.(39) Furthermore, MDSCs isolated from mice with myeloma had a greater capacity to form osteoclasts than MDSCs from control mice.(39) MDSCs were isolated from lacZ^+^ mice bearing myeloma. Following coinoculation of these lacZ^+^ MDSCs with myeloma cells into recipient mice, cells positive for both lacZ and TRACP were observed on the bone surface, demonstrating the MDSCs have the capacity to differentiate into osteoclasts in vivo.(39,40) What did this mean? It means that this population of MDSCs that are mobilized in vivo during initiation of the disease has multiple potentials, one of which is to progress down the osteoclast lineage to form mature bone-resorbing osteoclasts (Fig. 2). Future directions of research should be aimed at identifying the mechanisms by which myeloma cells induce these MDSCs to form cells of and to differentiate down the osteoclast lineage and to clarify the molecular mechanism by which they influence and possibly control myeloma cell function.

Myeloma serves as a potential paradigm for similar events occurring in solid tumors that metastasize to bone. In these situations, it is possible that the molecular mediators may be different, but the general principles may remain the same. In support of this, an increase in the proportion of granulocytic myeloid-derived suppressor cells, defined as CD11b^+^GR-1^+^Ly-C6^−^, was demonstrated in the long bones of a syngeneic mouse model of breast cancer bone metastasis.(41)

If these cells are so important to the skeletal complications associated with cancer, then one question is whether depletion of MDSCs affects tumor progression. There are potential mechanisms by which this could be achieved, for example, by liposome-mediated clodronate or other biosphonates, and it would be interesting to see the efficacy of these approaches when used in this manner. Bisphosphonates are in clinical use for the treatment of myeloma bone disease, and their effects on mature osteoclasts are well studied. However, little is known of their effects, if any, on osteoclast precursors such as MDSCs. Melani and colleagues demonstrated that zoledronate decreased the expansion of MDSCs associated with tumor-bearing mice.(42) This was mediated through inhibition of MMP-9. Our own studies have demonstrated that bisphosphonate treatment of myeloma-bearing mice results in a decrease in the proportion of MDSCs.(43) Isolation of these MDSCs from myeloma-bearing mice treated with zoledronate confirmed a reduction in their ability to form osteoclasts compared with untreated myeloma-bearing mice. No evidence of inhibition of protein prenylation was detected in MDSCs isolated from myeloma-bearing mice treated with zoledronate, suggesting that this effect of bisphosphonates on MDSCs is indirect and independent of the characteristic inhibition of protein prenylation in mature osteoclasts.

## Summary

MDSCs are a heterogeneous population of cells that are enhanced in tumor-bearing hosts and appear to play a number of roles in immune suppression and metastasis. It is exciting to speculate that these cells may have a distinct role in bone metastases, related not only to their immune suppressive properties but also to their potential to contribute to osteoclastogenesis and the subsequent destructive osteolytic bone disease. Future studies to elucidate their specific role in osteolytic bone disease not only will increase our understanding of the pathophysiology of cancer-induced bone disease but also may reveal novel therapeutic targets for the treatment of this destructive characteristic feature of bone metastases.
